# Finger Millet: A “Certain” Crop for an “Uncertain” Future and a Solution to Food Insecurity and Hidden Hunger under Stressful Environments

**DOI:** 10.3389/fpls.2017.00643

**Published:** 2017-04-25

**Authors:** Sanjay Mohan Gupta, Sandeep Arora, Neelofar Mirza, Anjali Pande, Charu Lata, Swati Puranik, J. Kumar, Anil Kumar

**Affiliations:** ^1^Molecular Biology and Genetic Engineering Laboratory, Defence Institute of Bio-Energy Research, Defence Research and Development OrganisationHaldwani, India; ^2^Department of Molecular Biology and Genetic Engineering, College of Basic Sciences and Humanities, G. B. Pant University of Agriculture and TechnologyPantnagar, India; ^3^Council of Scientific and Industrial Research-National Botanical Research InstituteLucknow, India; ^4^Institute of Biological, Environmental and Rural Sciences, Aberystwyth UniversityAberystwyth, UK; ^5^Department of Plant Pathology, College of Agriculture, G. B. Pant University of Agriculture and TechnologyPantnagar, India

**Keywords:** abiotic and biotic stress, genomics, finger millet, metabolomics, omics, phenomics, proteomics, transcriptomics

## Abstract

Crop growth and productivity has largely been vulnerable to various abiotic and biotic stresses that are only set to be compounded due to global climate change. Therefore developing improved varieties and designing newer approaches for crop improvement against stress tolerance have become a priority now-a-days. However, most of the crop improvement strategies are directed toward staple cereals such as rice, wheat, maize etc., whereas attention on minor cereals such as finger millet [*Eleusine coracana* (L.) Gaertn.] lags far behind. It is an important staple in several semi-arid and tropical regions of the world with excellent nutraceutical properties as well as ensuring food security in these areas even during harsh environment. This review highlights the importance of finger millet as a model nutraceutical crop. Progress and prospects in genetic manipulation for the development of abiotic and biotic stress tolerant varieties is also discussed. Although limited studies have been conducted for genetic improvement of finger millets, its nutritional significance in providing minerals, calories and protein makes it an ideal model for nutrition-agriculture research. Therefore, improved genetic manipulation of finger millets for resistance to both abiotic and biotic stresses, as well as for enhancing nutrient content will be very effective in millet improvement.

**Key message:** Apart from the excellent nutraceutical value of finger millet, its ability to tolerate various abiotic stresses and resist pathogens make it an excellent model for exploring vast genetic and genomic potential of this crop, which provide us a wide choice for developing strategies for making climate resilient staple crops.

## Introduction

Agriculture productivity is adversely affected with serious impact on production and productivity due to uneven weather conditions increased temperature and less availability of irrigation water. Global climate change together with the rapidly increasing population is mounting considerable pressure on agriculture sector to produce more food from less land. The anticipated increase in temperature will mostly affect the hot tropics, mainly populated by developing countries as they are likely to suffer maximum loss in food production (Cline, 2007). Even in temperate regions, several strategies need to be devised for the adaptation of agricultural crops against erratic climate conditions such as changing temperature, erratic rainfall, and onset of severe floods and droughts (Meehl et al., 2007). It has been anticipated that climate change may severely impact food production and food security in several drought-prone regions across the globe (FAO, 2005). This water paucity is leading to shrinking of dietary range and reduction of total food consumption that could possibly lead to malnutrition problems and food insecurity (Intergovernmental Panel on Climate Change [IPCC], 2007). A key issue is whether we will be able to feed the projected global population of 9 billion in 2050 equitably, healthily and sustainably (Beddington, 2010). Even if a person consumes enough calories, it is likely that he may have an inadequate consumption of vital micronutrients such as vitamins, minerals and trace elements leading to micronutrient undernourishment or what can be termed as hidden hunger. Pests and diseases are also likely to be greatly impacted by changing temperatures (Stireman et al., 2005).

Thus development of varieties with enhanced nutraceutical value and improved stress tolerance has been one of the priority areas of research these days. Modern crop improvement techniques such as genomics-assisted breeding and genetic engineering play important role in understanding the complexities of stress response and tolerance as well as in providing measures for enhanced crop productivity. However, one of the possible solutions to counter these tribulations can be identifying and improving native crops that are highly adaptive to local climate, have high nutritive value and can efficiently withstand biotic and/or abiotic stresses. Although it is difficult to find a single staple food crop that fulfills all the major criterions, the wide variety and diversity of local food crops (such as minor millets) provide us a choice of such climate resilient crops (Shukla et al., 2015).

## Millets: A General Introduction

Most world population depends upon cereals as their staple food. Wheat, rice and corn have been the preferred cereals, whereas millets have largely been neglected, more so in the aftermath of green revolution. Millets represent a diverse group of small-seeded grasses grown for food, feed or forage (Lata et al., 2013; Lata, 2015). They comprise about a dozen crop species that mainly originated in third world countries, and were domesticated and cultivated by small farmers in semi-arid and tropical regions. Distinctive attributes of the millets are their adaptation to adverse climatic conditions, requirement of minimal inputs, and superior nutritional properties (Lata et al., 2013). Millets are critical plant genetic resources for the agriculture that extends food security to deprived farmers inhabiting arid, infertile, marginal and poor lands especially in Asia and Africa. The fast maturation and all-season growth characteristics make them desirable crops for more intensive cropping systems and they could also be utilized as a catch or relay crop in combination with other crops that are slow in maturation. Despite the fact that they are staple in the diets of millions of people residing in the semi-arid and arid regions of the world, millets are sometimes referred to as “Orphan Crops,” or even “Lost Crops.” These crops are not actually lost but the term indicates their abundance by the developed countries and also their world production statistics indicate significantly low volumes compared to the other more popular food crops. However, these neglected crops are important by virtue of their contribution to biodiversity and the means of livelihood of the poor in various parts of the world (Belton and Taylor, 2004).

Millets comprised of six major small-grained cereal crops, namely finger millet (*Eleusine coracana*), foxtail millet (*Setaria italica*), kodo millet (*Paspalum scrobiculatum*), proso millet (*Panicum miliaceum*), barnyard millet (*Echinochloa* spp.), and little millet (*Panicum sumatrense*), and all of them are known for their unique traits and nutritional values (Kumar et al., 2016a,b). Millets need very little water for their production and can be cultivated under non-irrigated conditions or in very low rainfall regimes (200–500 mm). Single crops such as rice and wheat might provide food security but their cost of production remain high, while millets account for manifold securities including food, fodder, fiber, nutrition, health, environment and livelihood at minimal cost, making them the essential guardians of agricultural security.

Despite the well-documented health benefits of millets as excellent source of nutrients and minerals, they also contain some anti-nutrients (commonly called as phytochemicals) that negatively affect its nutrient values by reducing the digestibility of nutrients and mineral absorptions (Sarita, and Singh, 2016). These anti-nutrients mainly include phytates, polyphenols, oxalic acids, tannins and digestive enzyme inhibitors etc., may cause negative metabolic alterations (Singh and Sarita, 2016). For example phytic acid binds with dietary minerals such as Ca, Fe, Mg, and Zn and inhibits their absorption in our body. However, the negative impact of these anti-nutrients can be taken care by using common household food processing techniques like decortications, milling, soaking, malting, germination, fermentation, popping and cooking etc. Since, these methods can reduces the content of phytates, phenol, tannins and trypsin inhibitor activity and improve the digestibility of millets and also makes bioavailability of minerals (Shibairo et al., 2014).

## Finger Millet: An Economically Important Nutraceutical Crop

The generic name *Eleusine* is derived from the Greek goddess of cereals, “*Eleusine*” while the common name finger millet indicates “finger-like” branching of the panicle. As such, it may be one of the oldest indigenous domesticated tropical cereals in Africa. It is a highly productive crop that can thrive under a variety of harsh environmental conditions, and is also organic by default. It can be grown on low fertility soils and is not dependent on the use of chemical fertilizers, hence, is a boon for the vast arid and semi-arid regions (Gull et al., 2014). The different genotypes of finger millet have genes for early and vigorous growth, large panicle size, increased finger number and branching as well as high-density grains. Some of the genotypes are water-efficient with elevated carbon dioxide fixation rates and minimal leaf area and hence could perform extraordinary well in semi-arid climates. It is also known to be one of the most efficient utilizer of nitrogen (Gupta et al., 2014). Finger millet seeds can resist storage pests for as long as 10 years, ensuring round the year food supply or even during a crop failure, that has earned it the popular name of ‘famine crop’ (Mgonja et al., 2007).

Finger millet is a crucial for the diets of pregnant and lactating mothers, and children as well for the economy of marginal farmers. Its grains are rich in protein, vitamins, minerals, fiber content and energy as compared to other cereals (Vadivoo et al., 1998). Some genotypes of finger millet have been analyzed to contain calcium as high as 450 mg/100g of grains (Gupta et al., 2011; Kumar et al., 2014c) and hence, can be developed and used as preventive drug(s) against osteoporosis. It is also enriched with manganese, phosphorus and iron as well as useful amounts of copper and comparatively higher chromium, magnesium, molybdenum, zinc and selenium (Shashi et al., 2007; Tripathi and Platel, 2010). Also, finger millet straw is excellent as animal fodder with up to a total of 60% digestible nutrients.

Its seed coat is rich in phytochemicals like dietary fiber and polyphenols and is also very high in minerals especially calcium (Devi et al., 2014). Chethan and Malleshi (2007) showed upto 2.3 ± 0.3 gallic acid equivalents in whole meal and upto 6.4 ± 1.5 in the seed coat of finger millet grains. The seed coat also shows anti-cancer and anti-diabetic activities, mainly due to its high polyphenol content that indicates anti-oxidant activity, and high fiber that promotes slow digestion and blood sugar stability (Devi et al., 2014). Therefore, finger millet has maintained high socio-economic importance in the context of subsistence farmers of the Indian and African semi-arid tropic regions (Gull et al., 2014).

Finger millet contains amino acids in concentrations exceeding those of FAO/WHO recommended standards. It has high levels of methionine and lysine (Mbithi-Mwikya et al., 2000), which are lacking in the diets based on starchy foods. The finger millet grains are superior to rice and wheat as it contains essential amino acids such as methionine and tryptophan, (Fernandez et al., 2003). Supplementation of finger millet with lysine, proteins or legumes such as green gram, soybeans and chickpeas was found to improve its protein quality even further (Belton and Taylor, 2004). Sridhar and Lakshminarayana (1994) compared the lipid content and composition of foxtail millet, proso millet, and finger millet grains and found that finger millet contained triacylglycerol accounted for 80% of the total lipid, while phospholipid and glycolipid accounted for 14 and 6% of the total lipid, respectively. Phosphatidylglycerol, phosphatidylethanolamine, phosphatidylcholine and digalactaosyl monoglycerides predominated in the phospholipid and glycolipid fractions. The concentration of digalactosyl monoglyceride was highest in finger millet among the three millets studied. Also, finger millet lipid contained more palmitic acid as compared to other millets.

Finger millet is appreciated for its slow digestibility thereby furnishing energy throughout the day. The plant itself is reported to be diaphoretic, diuretic, and vermifuge, and its leaf juice has been given to women in childbirth (Dida and Devos, 2006). It has also been used as a folk remedy for various ailments including leprosy, liver disease, measles, pleurisy, pneumonia, and small pox (Dida and Devos, 2006). The high fiber content of finger millet helps in preventing constipation, high cholesterol formation, diabetes and intestinal cancer (Devi et al., 2014). Kumari and Sumathi (2002) also suggested significantly lower plasma glucose levels after consumption of finger millet based diets as compared to those based on rice and wheat, due to higher fiber content. However, the presence of some anti-nutritional factors in whole finger millet flour that could reduce starch digestibility and absorption may be responsible for the lower glycemic response (Shobana et al., 2007). Studies conducted by Pradhan et al. (2010) on male and female diabetic patients spread across different rural and urban locations, also showed that the glucose level was maintained after consuming multigrain chapati of finger millet and wheat in a ratio of 30:70. Another study by Nazni et al. (2010) showed the positive impact of weaning biscuits supplements on the nutritional aspects and cognitive functions of children. Finger millet is gluten free and hence can be a boon for patients suffering from celiac disease (Pagano, 2006) as a strict gluten-free diet is currently the only treatment for this disease. Also, the risk of diabetes and gastrointestinal tract ailments could be effectively reduced with regular consumption of finger millet.

## Finger Millet-Organic By Default: Transcriptional Regulation of Nitrogen Use Efficiency

Cereal crops use less than half of the applied nitrogenous fertilizers indicating low NUE. It indicates that disproportionate use of nitrogenous fertilizers goes waste. This unusable nitrogen is fast becoming an environmental threat. As the ‘second green revolution’ emphasizes mainly on the technologies that can increase yields without damaging the environment, therefore, research efforts need to be focused to develop crop varieties with high NUE under low nitrogen supplementation. In view of the fact that, common cereal genomes have limited gene pool for high NUE trait, an understanding on the mechanism of high NUE of finger millet seems to be most fitting for agricultural research, as it can thrive on almost no nitrogen inputs yet accumulates high quality proteins enriched with essential amino acids in their grains. This strongly indicates that finger millet has developed unique mechanisms of utilizing available soil nitrogen that also enables the plant to achieve its full yield potential (Kumar A. et al., 2009; Kumar R. et al., 2009). Some non-pathogenic bacteria and fungi found in plant tissue commonly called as symbiotic endophytes are also reported in finger millets, which are responsible for nitrogen fixation and make nitrogen available to plants even at no nitrogen supplementation (Goron et al., 2015). However, the detailed mechanism by, which finger millet achieves this extraordinary feature is still poorly understood.

Gupta et al. (2011) investigated the possible mechanism of high NUE in finger millet, through studies on growth, yield and NUE components besides the activities of important enzymes in various genotypes. These studies suggested that grain protein content was inversely correlated with NUE and nitrogen utilization efficiency but positively correlated with nitrogen uptake efficiency. NUE in plants is a complex trait and hence it involves the participation of many genes that are involved in nitrogen uptake, assimilation and distribution within the plant. Moreover, it is also intimately linked with carbon metabolism. Since manipulating large number of genes to achieve high NUE appears to be practically impossible, identification of novel nitrogen responsive genes and their *cis* and *trans* acting elements becomes a necessity. Incidentally, many of the promoters of genes involved in carbon and nitrogen metabolism have been found to contain DNA binding sites recognized by a plant specific transcription factor known as Dof1 which has been shown to act not only a master regulator of genes involved in carbon and nitrogen metabolism but also plays important role in the regulation of NUE in plants (Kushwaha et al., 2008; Kumar A. et al., 2009; Kumar R. et al., 2009; Kumar et al., 2014b). It has been showed that FmDof1 expression is higher in brown (PRM-1) and golden (PRM-701) genotypes as compared to white (PRM-801) genotype, which corroborates that Dof1 has a major role in nitrogen assimilation (Kumar R. et al., 2009b; Gupta et al., 2011). Parallel to this, *in silico* analysis and wet experiments were performed in rice and finger millet to understand differential nitrogen responsiveness (Gaur et al., 2011, 2012a,b). These results suggests, at low nitrogen condition the high grain protein content genotype is high nitrogen responsive compared to the low grain protein content genotypes (Gupta et al., 2011, 2013; Kumar et al., 2013). In order to understand the molecular mechanism of NUE and how Dof1 regulate genes of carbon and nitrogen metabolism, the expression of FmDof1 was studied under different light-dark conditions in two genotypes of finger millet. Results depicts that *Dof1* expression in higher grain protein genotype was more consistent with that of the carbon and nitrogen metabolism genes suggesting that it differentially regulated the expression of these genes and also simultaneously controlled the grain protein content in finger millet (Gupta et al., 2013; Kanwal et al., 2014). Recently, the transcriptome data generated led to discovery of numerous genes expressed during spike development in finger millet (Kumar et al., 2015a). Expression analysis of 10 *Dof* genes in two contrasting finger millet genotypes was differential further, suggesting their role during accumulation of seed protein.

As finger millet has become a model system to understand how nitrogen is efficiently utilized even under low nitrogen conditions. Some glimpses and significant outcome in this area has been achieved as discussed above. However, the functional validation of FmDof1 and FmPBF Dof genes and their synergistic role would further provide useful insights into understanding the mechanisms of grain filling and high NUE in finger millet.

## Abiotic Stress Tolerance in Finger Millet

Abiotic stresses are grave threats to the global food security as crop productivity and geographical distribution of the crops for agriculture are adversely affected causing significant economic losses. The degree of susceptibility to abiotic stresses varies from species to species (Dida and Devos, 2006). Adaptability to stress is a complex phenomenon that is regulated at several levels including physiological, cellular and molecular. Delineating the mechanisms of plant stress tolerance and adaptation has long been the area of interest to agricultural scientists. Finger millet is one of the highly valued crops for its ability to grow under limited resources and also for its high nutrient contents (Gull et al., 2014). Despite these unique characteristics, it has been neglected for many years. Fortunately, this species has preserved its biodiversity. Global concerns over malnutrition, food insecurity and loss in agricultural productivity due to uncertain climatic changes have resulted in an increased demand for climate resilient crops. According to the World Summit on Food Security at least 70% more food production is required by 2050 to feed the ever increasing population. It would require annual increases of approximately 44 million tons, which is 38% above current annual increases in food production (Tester and Langridge, 2010). Under this scenario, finger millet has gained focus of scientific research for their extraordinary potential to grow under high temperature, low moisture and poor soils.

Though finger millet is considered to be generally abiotic stress tolerant, even then there is a need to identify newer sources of stress tolerance in this crop that can be used for crop improvement programs. Assessing patterns of genetic diversity in terms of both abiotic and biotic stress tolerance in finger millet germplasm collections could be very critical. A large number of finger millet accessions have been preserved in various national and international GenBanks in Asian, African, and European countries as well as USA (**Table 1**). India has the largest collection of finger millet germplasm followed by Ethiopia (Dwivedi et al., 2012). There could be immense morphological and genetic diversity among finger millet accessions or their core collections. Molecular markers could be used to characterize functional diversity in this crop (Kumar et al., 2015c). Calcium dynamics (Yadav et al., 2014), tryptophan accumulation and association mapping (Babu et al., 2014a,b), and disease resistance (Babu et al., 2014c) have been characterized using molecular markers (Babu et al., 2014e). However, characterization of abiotic stress tolerance in finger millet using molecular markers is yet very limited and offers an opportunity of exploring the vast collections of wild and cultivated accessions of this crop.

**Table 1:** Number of worldwide significant cultivated germplasm collection of finger millet preserved in national and international gene banks.

Country	Institutes	No. of accessions	Reference
**Asia**			
India	All India Coordinated Millet Project, UAS, Bangalore	6257	Dwivedi et al., 2012
	International Crop Research Institute for the Semiarid Tropics (ICRISAT), Patancheru	6804	Goron and Raizada, 2015
	National Bureau of Plant Genetic Resources (NBPGR), New Delhi	9522	Dwivedi et al., 2012
Japan	Department of Genetic Resources I, National Institute of Agrobiological Sciences (NIAS), Tsukuba-shi	565	Dwivedi et al., 2012
Nepal	Central Plant Breed. and Biotechnol. Division, Nepal Agric. Res. Council (CPBBD), Khumaltar, Kathmandu	869	Dwivedi et al., 2012
**Africa**			
	Ethiopia Institute of Biodiversity Conservation, Addis Ababa	2156	Dwivedi et al., 2012
Kenya	National Gene Bank of Kenya, Crop Plant Genetic Resources Centre, Muguga	2875	Dwivedi et al., 2012
Uganda	Serere Agricultural and Animal Production Research Institute, Soroti	1231	Dwivedi et al., 2012
Zambia	SADC Plant Genet. Resour. Centre, Lusaka	1037	Dwivedi et al., 2012
**America**			
USA	National Center for Genetic Resources Preservation, Fort Collins, Colorado, USA	702	Dwivedi et al., 2012
	Plant Genetic Resources Conservation Unit, USDA-ARS, Griffin, GA, USA	748	Dwivedi et al., 2012

### Delineating Abiotic Stress Tolerance Mechanism in Finger Millet

With the advent of the science of *omics* and high throughput sequencing technologies, it has become evident that stress regulation is a highly complex and inter-reliant phenomenon and is crucial for a plants survival under sub-optimal conditions. It has been clearly demarcated that stress tolerance involves a network of regulatory and signaling molecules, which may function synergistically or antagonistically. Millets with their highly adaptive traits are a treasure trough of important genes and regulatory proteins that can be exploited to develop stress resistant crops. Amongst the small millets, finger millet is the most climate resilient crop, which can be grown under a wide spectrum of extreme climatic conditions. Thus, they can be termed as “farmer friendly” crops providing them better returns in comparison to other crops, which are subjected to changing climatic conditions. Moreover, from a breeder’s point of view, these are the source of traits which can improve hardiness of other widely grown cereals. From biotechnological point of view its “hardy” nature is of great interest to the researchers worldwide, exploring it and exploiting it for developing resilience in other economically important crops (Kumar et al., 2015b). In order to fully understand and appreciate the abiotic stress tolerance potential of finger millet, the language of their DNA needs to be deciphered. Here we present the recent genomics and proteomics approaches for exploiting the genetic potential of finger millet for developing resilience in other crops and enhancing their productivity under sub-optimal growth conditions.

#### Genomics Approaches

**FIGURE 1:**
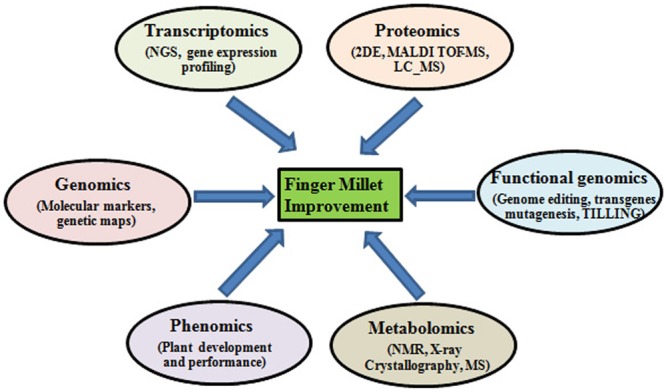
**Different omics approaches for improving abiotic and biotic stress tolerance in finger millet**.

The genetic potential of finger millet serves as an indispensable resource for understanding the genomics of abiotic stress tolerance (Kumar et al., 2015b). Genomic approaches like structural, functional and comparative genomic can be utilized to determine the unique traits found in finger millets and exploit them for crop improvement purposes (**Figure 1**). Functional genomics has led to the identification of several nutritionally important genes including those encoding calcium transporters and seed storage proteins. *De novo* RNA sequencing technology has been used to sequence the finger millet grain-filling stages transcriptome (Kumar et al., 2015a). The initial genome assembly of finger millet (1593 Mbp; chromosome no. 2n = 36) has already been completed and the full sequence is expected to be published soon by the Bio-resources Innovations Network for Eastern Africa Development (Bio-Innovate). Once the complete genome is assembled, genome annotation will serve to determine the regions of the code with novel genes. This will enable scientists to locate the key genes in the genome playing role in abiotic stress tolerance and also other useful genes for crop improvement purposes.

Comparison of the genomes across species can reveal the similarities and differences in genome structure and organization (comparative genomics). Such studies can reveal the evolutionary relationship between species and may be useful in predicting key genes playing role in abiotic stress tolerance. Indeed, extensive similarities have been known to be shared across plant genomes of even distantly related species (Guyot et al., 2012). Orthologous sequences in the aligned genomes can be analyzed for the extent of conserved sites. Comparative genomics helps in determining a virtual gene order in a partially sequenced genome with the emergence of the concept of “genome zipper,” which basically compare the fully sequenced and annotated genomes with various sources of data derived from less well-studied species (Mayer et al., 2011). Together, structural and functional genomics can help in characterizing a genome fully. Candidate transcription factors (TFs) genes with desirable traits such as stress tolerance can be identified with the help of genome wide expression profiling. Further inactivation or over-expression studies of these stress responsive TFs genes can be done for the development of transgenic crop with desired traits.

The genetic improvement of finger millet through transgenic technology has, however, been inadequate regardless of its significance as a nutraceutical crop and also that improved stress tolerance along with superior grain yield has been a priority area of research for this crop (Kumar et al., 2014a; Lata, 2015). The two earlier reports on finger millet transformation were through biolistic method (Gupta et al., 2001; Latha et al., 2005). *Agrobacterium-*mediated transformation of finger millet using shoot explants was established by Ceasar and Ignacimuthu (2011). There has been only one report on generation of transgenic finger millet with improved multiple stress tolerance including drought, salinity and oxidative stress (Hema et al., 2014). In this study the tolerance in finger millet was achieved through the stable expression of *mtlD* gene from bacteria. Further, efforts have been made to understand the role of monodehydroascorbate reductase, a key anti-oxidant enzyme, in finger millet under different abiotic stresses (Sudan et al., 2015). Krishnamurthy et al. (2014) identified salt tolerant finger millet germplasms. The drought tolerant genotypes (PRM6107 and PR202) of finger millet may be used for allele mining of drought responsive genes for developing transgenic varieties using biotechnological tools (Bartwal et al., 2016). In this effort, over-expression of a finger millet TF gene, *EcNAC1*, in tobacco conferred abiotic stress-tolerance (Ramegowda et al., 2012). Recently, Rahman et al. (2016) also showed expression of an *EcNAC67* TF from finger millet imparted salt and drought tolerance in rice. There have also been a few reports of transcriptome analyses in finger millet highlighting its mechanisms for salinity stress tolerance (Rahman et al., 2014) and calcium accumulation (Singh U.M. et al., 2014). Since, finger millet is known as a hardy nutritionally important crop, it can be argued that for maximal exploitation of its genomes for abiotic stress tolerance, the genomics approach is indispensable (**Table 2**).

**Table 2:** Genetic manipulations for improving abiotic and biotic stress tolerance in finger millet.

Gene name	Source of the gene	Stress tolerance	Reference
EcDehydrin7	Finger millet	Over expression of EcDehydrin7 induce abiotic stress tolerances	Singh R.K. et al., 2014
Ec-apx1	Finger millet	Expression increased under drought	Bhatt et al., 2013
Metallothionein	Finger millet	Induced under drought	Parvathi et al., 2013
Farnesylated protein ATFP6	Finger millet	Induced under drought	Parvathi et al., 2013
Farnesyl pyrophosphate synthase	Finger millet	Induced under drought	Parvathi et al., 2013
Protein phosphatase 2A	Finger millet	Induced under drought	Parvathi et al., 2013
RISBZ4	Finger millet	Induced under drought	Parvathi et al., 2013
NAC 67	Finger millet	Tolerance against salinity and drought stress in rice	Rahman et al., 2016
mtlD	Bacteria	Over expression induced multiple stress tolerance	Hema et al., 2014
monodehydroascorbate reductase	Finger millet	Over expression induced drought, salt and UV radiation tolerance	Sudan et al., 2015
C2H2 type of zinc finger transcription factors (TFs)	foxtail millet	Salinity, dehydration and cold stress	Muthamilarasan et al., 2014
EcNAC1	Finger millet	Abiotic stress tolerance	Ramegowda et al., 2012
EcJAZ	Finger millet	Over expression induces abiotic and biotic stress tolerance	Sen et al., 2016

#### Proteomics Approaches

Proteomics is another important functional genomic approach that has been demonstrated its utility in identifying novel stress responsive proteins that could be exploited for improving stress tolerance of important agricultural crops. Under stress various ion transporters and signaling cascades and regulatory proteins are activated, knowledge of the proteins involved in these processes can give insights into the unique characteristics of small millets that can be exploited for crop improvement (**Figure 1**). Komatsu and Hossain (2013) underlined the necessity for organ-specific proteomic analyses for identifying proteins that are usually accumulated in various plant organs and intracellular compartments under various abiotic stresses and thus may play a dominant role in plant stress responses (Komatsu and Hossain, 2013).

The application of the emerging proteomic technology comprising MS has increased accuracy and throughput. Jacoby et al. (2013) have provided a comprehensive method to rank the relatively important stress-responsive proteins. Advancements in MS platforms has brought a new revolution in proteomics as it has become an crucial tool for investigating the post translational modifications (PTMs) to proteins, and protein–protein interactions giving insights of cellular processes (Cox and Mann, 2007). LC-based proteomice analyses is also becoming increasingly common in several laboratories. However, the application of crop proteomics has largely been slowed down due to the limited availability of genomic information. So far, proteomics techniques have not yielded much information regarding the stress tolerance potential of small millets particularly finger millet. One of the probable reasons being lack of genomic data, yet advances in research continues to build up new hopes toward reaching the goal of sustainable agriculture.

## Biotic Stress Tolerance in Finger Millet

Finger millet is known to be protected from diseases for decades and several blast resistant lines of finger millet have been identified in the past 15 years to identify the source of immunity (Ramappa et al., 2002). Both the physical and chemical composition of the grains determines the mechanisms of resistance to pests and pathogens. The physical structure of the grain acts as the first line of defense against infection and infestation. Small size and grain hardness is a major restraint that has been found to reduce mold infestation (Audilakshmi et al., 1999). Apart from this, composition of the cell walls (Kavitha and Chandrashekar, 1992), pigmented testa, seed phenols (such as ferulic acid) and glume color contribute to grain mold resistance (Audilakshmi et al., 1999). For example, Seetharam and Ravikumar (1993) reported significantly higher quantity of total phenols in brown colored grains (resistant cultivars) than whites (susceptible cultivars). Similarly, Chethan and Malleshi (2007) have reviewed the role of polyphenols such as flavonoid and p-coumaric acid in finger millet. These correlation studies between blast disease and phenols indicated strong negative association. In addition, plants usually respond to fungal infection by producing a variety of toxic compounds known as phytoalexins, which may be one of the several ways toward extending defense capability of the grains (Snyder et al., 1991).

Several bioactive and AFPs that are induced in response to pathogen attack have been identified and characterized in millets during the past few years (Gatehouse and Gatehouse, 1998). Prolamins are the grain storage proteins of finger millet and are structured into protein bodies that serve as a physical and a nutritional barrier due to their resistance against digestion by the insect and fungal proteases (Gupta et al., 2011). A surplus of PR proteins present in the grain, some of them located in protein bodies are also important in containing infestation. Apart from this, millets, cereals and leguminous plants also produce several proteinaceous enzyme inhibitors that act on crucial digestive hydrolases of the insect gut. For example, the α-amylases and proteinases regulate a number of phytophagous insects (Gatehouse and Gatehouse, 1998). The only inhibitor possessing both functions is the α-amylase/trypsin inhibitor (RBI), which is bifunctional and has been isolated from finger millet and extensively studied (Strobl et al., 1995). Later on, ammonium sulfate fractions derived from the finger millet grain extracts has been tested against α-amylases of several storage insects and other insect pests, and the results indicated the extent of inhibition by the different insects varied from 8.0 to 69.9% with the highest inhibition (69.9%) against the *Callosobruchus chinensis* (the pulse beetle) α-amylase (Payan, 2004; Sivakumar et al., 2006). Similarly, Sen and Dutta (2012) cloned and expressed the bifunctional inhibitor (RBI) in *Escherichia coli* Rosetta2 (DE3) cells and reported a significant increase in *rbi* transcript accumulation in leaves of finger millet when infected with *Rhizopus oryzae* and *Curvularia lunata*.

Blast disease caused by *Magnaporthe grisea* (anamorph *Pyricularia grisea*) is one of the major limiting factors for the production and productivity in finger millets. Identification of QTLs/genes linked to important physiological traits such as blast resistance will be useful for molecular breeders to introgress those genes into locally well adapted germplasm. In this effort, molecular markers linked to blast resistance were identified, which could be used to develop blast resistant genotypes through marker assisted selection (Panwar et al., 2011). Later on, it was found that high levels of synteny existed between NBS-LRR regions of finger millet and rice and were found to be on nearly same positions when mapped on the rice chromosome map. A total of 8, out of 15 ESTs-based SSR primers were polymorphic among the selected resistant and susceptible finger millet genotypes, which when sequenced were found similar to the characteristic kinase-2 and kinase-3a motifs of rice and finger millet R-genes (Babu et al., 2014e). Also, a total of 58 genic SSR markers were derived from the EST sequences of different blast genes of rice by using comparative genomics approach for genetic analysis of blast resistance. These 58 SSR markers could group the 190 finger millet genotypes into four major clusters based on their response to blast disease (Babu et al., 2014d). A good correspondence between the phylogenetic tree, PCA analysis and the population structure could be observed differentiating the different finger millet genotypes into HS-MR, MR-R and R-HR clusters based on their blast disease response (Babu et al., 2014c). Five significant QTLs for finger blast and neck blast could be identified based on the association of SSR marker data with the leaf blast, neck blast and finger blast data. The QTLs for finger blast showed strong associated with the genic SSR primer FMBLEST32, which was designed from the rice blast gene Pi5 known for its road spectrum resistance to *M. grisea* and RM262, rice SSR. Similarly, the association mapping using MLM of structure software resulted in the identification of seven QTLs (three for finger blast, three for leaf blast and one for neck blast). Likewise, FMBLEST32 and RM262 were found to be associated to all finger millet blast diseases by both the approaches. The SSR marker UGEP53 was associated to finger blast by explaining the 10.5% of phenotypic variance (Babu et al., 2014b). The results obtained from association mapping showed that finger blast and neck blast resistant genes of finger millet were located on 2nd and 6th chromosomes suggesting them to be the major hub of blast resistant genes (Babu et al., 2014b). The identified markers can be further be utilized for fine mapping, full length blast genes cloning, and marker assisted breeding (MAB) programs of finger millet.

Advances in recombinant DNA technology and molecular tools have remarkably assisted in establish finger millet as a model to understand plant–microbe interactions. The initial work on finger millet transformation was carried out by Gupta et al. (2001) who compared the efficiency of five gene promoters that may be used for the elevated expression of candidate genes in finger millet. Later on, Latha et al. (2005) developed a transgenic finger millet resistant to fungal blast disease by chemically synthesizing the prawn antifungal protein (PIN), cloning and transforming it into finger millet plants that exhibited improved resistance to leaf blast disease. Later on, identification and characterization of chitinases in finger millets have revealed that their over-expression can combat fungal pathogens (Cletus et al., 2013). As hormone signaling plays a central role in plant defense against fungus and bacteria. Efforts have been made to understand the role of Jasmonate ZIM-domain (JAZ) protein family, in the regulation of JA signaling pathway (Sen et al., 2016) (**Table 2**). Thus, the genetic improvement of finger millet for increased grain yield by imparting resistance to fungal diseases and other biotic stresses is a priority. Understanding the molecular mechanisms of resistance in the finger millet grain will play key role in developing robust resistance against various pests and pathogens. One of the major constraints toward developing increased resistance to pathogens has been the relatively low level of resistance obtained when a single plant antifungal gene is used. The obvious strategy thus is to identify the combinations of such genes that will provide resistance against a broad spectrum of pathogens.

## Conclusion and Future Perspectives

Finger millet is no more called a *coarse cereal* rather referred as a nutri-cereal or as a nutraceutical crop and is seen as a potential solution for malnutrition and hidden hunger worldwide. Apart from its excellent nutritional value, its ability to tolerate various abiotic stresses and resist pathogens make it an excellent model for exploring vast genetic and genomic potential of this otherwise important crop and related cereal grasses. These properties thus on the whole make finger millet an ideal model for studying genomics and a plausible source for gene mining for complex traits.

Molecular biology and biotechnology has proved to be a promising tool for imparting stress tolerance in economically important plants, however, until now the progress is limited among millets mainly due to lack of appropriate genomic resources in these crops. However, with the availability of sorghum, foxtail millet and *Brachypodium* genome sequence, and on-going genomics program in finger millet and pearl millet will be of great help for the abiotic and biotic stress tolerance research in these minor cereals. High throughout sequencing platforms will not only be able to overcome the complexity of large and complex finger millet genome but will also help to understand the regulation of stress tolerance at transcriptional, post-transcriptional and epigenetic levels. An integration of various advanced high throughput omics strategies will definitely revolutionize finger millet research with the large-scale identification of stress responsive genes/proteins/metabolites that could potentially be used for crop improvement. Potential candidate genes responsible for high yield, biotic and abiotic stress tolerance and those involved in high mineral accumulation isolated from finger millet can also be utilized for improving other cereal crops through transgenic approaches or genomics-assisted breeding and pave way for the development of designer crops for a better and sustainable future. Production of transgenic crops expressing functional foreign genes has to be expanded to millets as well in order to produce transgenic finger millet varieties expressing foreign genes of agronomic importance, which will be very helpful in improving millet production by conferring resistance to both biotic and abiotic stresses. Development of a *super cereal* in the future may also be possible by incorporating various agronomically important traits into the genome of a single finger millet genotype. Thus utilization of current advances in molecular breeding and genetic engineering together with advanced *Omics* technologies will definitely prove useful in improving the present scenario of research in finger millet.

## Author Contributions

AK conceptualized the manuscript. SMG, SA, NM, and CL wrote the manuscript. AP assisted and CL, SP and JK edited the manuscript. SMG and AK contributed in critically revising the draft and updating the manuscript for publication. All authors read and approved the manuscript.

## Conflict of Interest Statement

The authors declare that the research was conducted in the absence of any commercial or financial relationships that could be construed as a potential conflict of interest.

## Abbreviations

2-DE: two-dimensional electrophoresis
AFLP: Amplified fragment length polymorphism
AFPs: antifungal proteins
ESTs: expressed sequence tags
GDH: glutamate dehydrogenase
GOGAT: glutamine oxoglutarate aminotransferase
GS: glutamine synthetase
JA: jasmonic acid
LC: liquid chromatography
MPSS: massively parallel signature sequencing
MS: mass spectrometry
mtlD: mannitol-1-phosphate dehydrogenase
NR: nitrate reductase
NUE: nitrogen use efficiency
PR proteins: pathogenesis related proteins
RFLP: restriction fragment length polymorphism
SAGE: serial analysis of gene expression
SNP: single nucleotide polymorphism
SSRs: simple sequence repeats
TILLING: targeting induced local lesions in genomes
